# Armchair MoS_2_ nanoribbons turned into half metals through deposition of transition-metal and Si atomic chains

**DOI:** 10.1038/s41598-018-31684-z

**Published:** 2018-09-06

**Authors:** Chi-Hsuan Lee, Joy Lin, Chih-Kai Yang

**Affiliations:** Graduate Institute of Applied Physics, National Chengchi University, Taipei, 11605 Taiwan Republic of China

## Abstract

MoS_2_ nanoribbons with armchair-terminated edges are semiconductors suitable for the tuning of electronic and magnetic properties. Our first-principles density function calculations reveal that a variety of transition-metal atomic chains deposited on some of the ribbons is able to transform the semiconductors into half metals, allowing transport of 100% spin-polarized currents. Furthermore, we found that a Si atomic chain is equally capable of achieving half metallicity when adsorbed on the same nanoribbon. These results should be useful for spintronic application.

## Introduction

Monolayers of transition-metal dichalcogenides^1^ possess tremendous potential for wide-ranging applications in the field of electronics. The MoS_2_ monolayer^2,3^, being one important member, is a semiconductor with a direct band gap around 1.8 eV and is well known to be associated with symmetry-related physical properties and optoelectronic application^4,5^ in the emergent field of valleytronics^6,7^. This moderate size of band gap is also very suitable for manipulation and tuning by a variety of methods for fabricating specialized electronic devices. One-dimensional circuits, for example, can be made of tailored MoS_2_ nanoribbons with specific types of edges and their associated band structures. Currently, synthesis of MoS_2_ monolayers, nanosheets, or nanoribbons can be accomplished in various ways involving chemical^8^ or mechanical exfoliation^9^, using chemical vapor deposition^10,11^, atomic layer deposition^12^, polymer-assisted deposition^13^, or a vapor–solid–solid mode^14^. MoS_2_ nanoribbons encapsulated in carbon nanotubes^15^ can have uniform widths as narrow as 1–4 nm and layer numbers down to 1–3.

In this article we concentrate on one type of MoS_2_ nanoribbons—those with armchair–terminated edges—and discuss their transformation as a result of the adsorption of transition-metal and silicon atomic chains. It will be shown that under certain circumstances various adsorbed atomic chains are capable of turning the semiconducting ribbon into a half metal, making the transport of electrons completely spin-polarized. This property provides a reliable conduit for spin transport and could further expand the application of MoS_2_ into the field of spintronics^16^.

**Figure 1:**
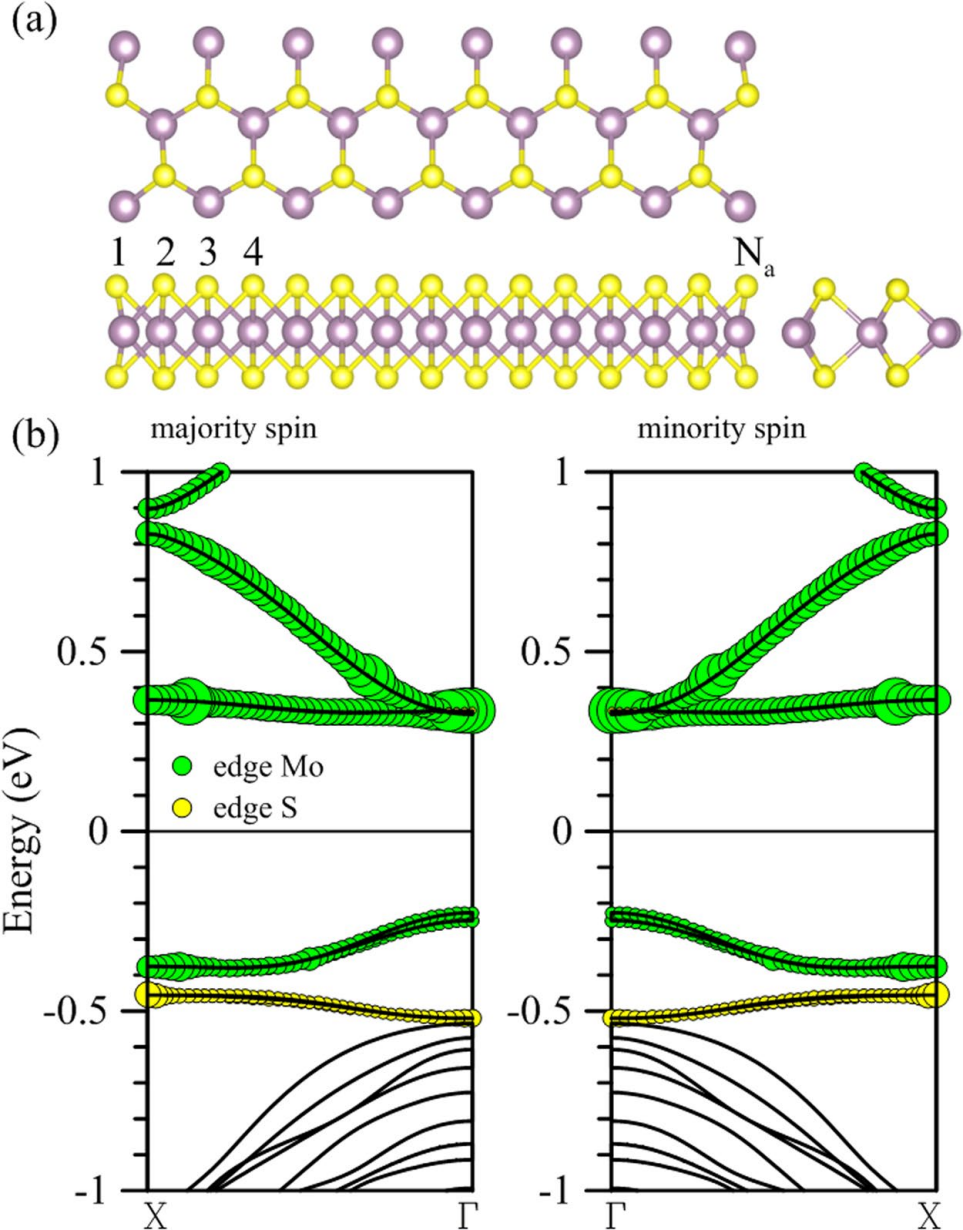
(**a**) A *N*a-MoS_2_ armchair nanoribbon with *N*a = 15. (**b**) Within the energy window between −1 and 1 eV, one valence band and all conduction bands are dominated by the contribution from edge Mo atoms, which are represented by the size of green circles. The band marked by yellow circles consists mostly of *p* orbitals from the edge S atoms.

A typical *N*a-MoS_2_ nanoribbon with armchair-terminated edges and *N*a denoting the ribbon width is shown schematically in Fig. 1(a) for a fully relaxed 15-MoS_2_ configuration in the x-y plane. Also shown is how S and Mo atoms are bonded along the z axis. The calculated Bond length between Mo and S atoms in the middle part of the ribbon ranges from 2.409 to 2.414 Å. At the ribbon edges, S atoms move outward and Mo inward slightly, rendering a shorter bond length of 2.292 Å between the Mo and S atoms. Spin-polarized energy bands are shown in Fig. 1(b) between −1 and 1 eV. Lower conduction bands and the uppermost valence band consist mostly of *d* orbitals from the edge Mo atoms, with edge S atoms contributing overwhelmingly to another valence band right below. A direct energy gap of 0.552 eV is also observed from the band structure, providing opportunities for manipulating the transport and optoelectronic properties. These results are very consistent with previous calculations^17–20^.

**Figure 2:**
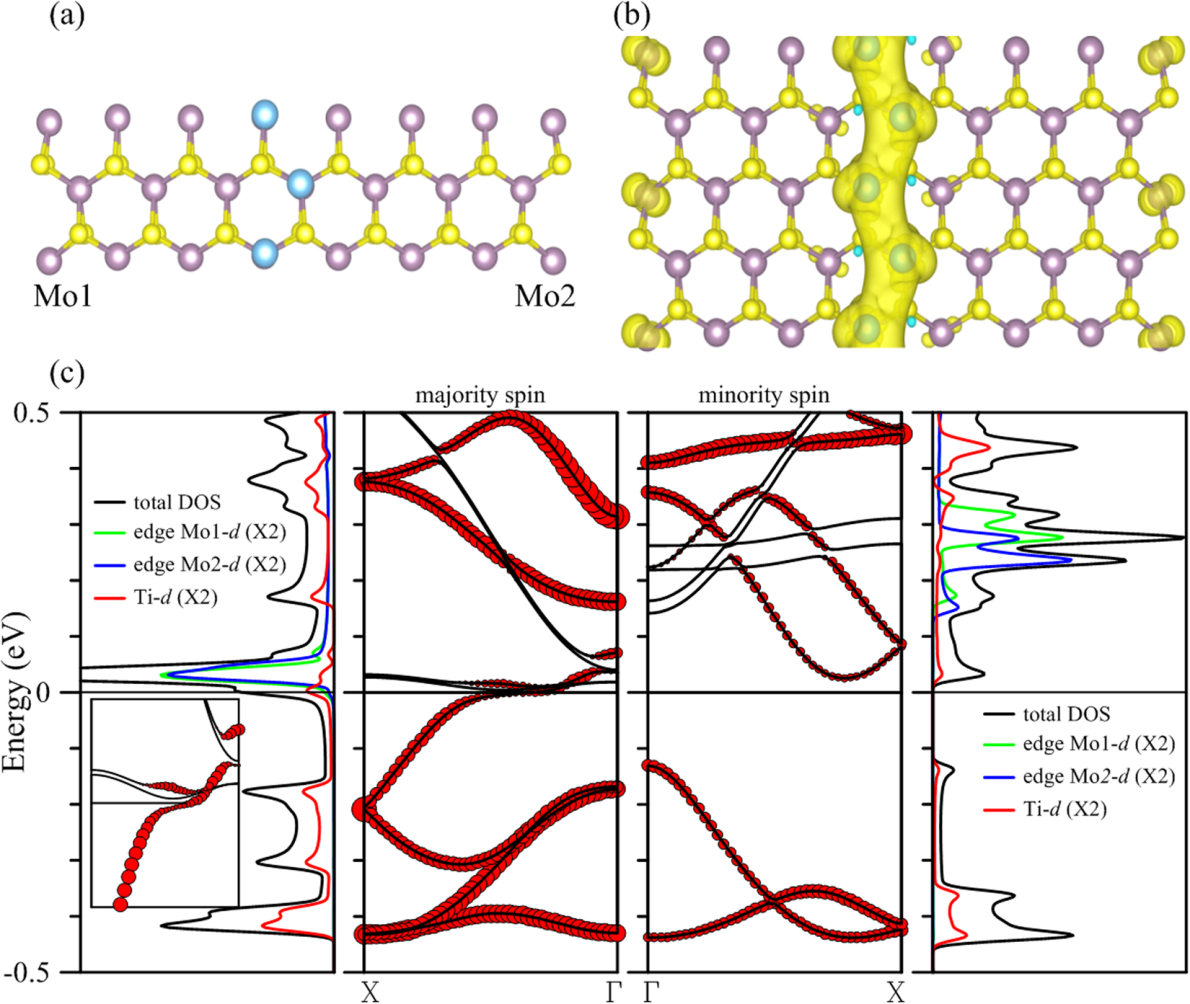
(**a**) Configuration of a single chain of Ti atoms (azure spheres) deposited in the middle part of the 15-MoS_2_ armchair nanoribbon, and (**b**) spin density of the composite. (**c**) The left (right) two panels are DOS and energy bands corresponding to the majority (minority) spin. Inset in the first panel is for bands in the second panel that are within 0.1 eV of the Fermi level. Local DOS of individual Ti and Mo atoms at both edges are magnified twice as large for clarity. Contributions of Ti atoms are marked by the size of red circles in the energy bands.

Next we consider Ti atoms forming an atomic chain over the 15-MoS2 armchair nanoribbon. A fully relaxed structure is shown in Fig. 2(a), which contains two Ti atoms in the unit cell. The chain winds its way through the middle part of the ribbon with Ti atoms directly atop Mo atoms. Binding between Ti atoms and their host is very strong. We calculated the binding energy per unit cell as the difference between the total energy of the Ti-deposited MoS_2_ ribbon and the combined total energies of the pristine ribbon and the two isolated Ti atoms and found the number to be −6.74 eV per unit cell (or −3.27 eV per atom), which is listed in Table 1.

**Table 1:** Binding energy and magnetic moment per unit cell for each of the three Ti-deposited 15-MoS2 armchair nanoribbons: a single center-deposited Ti chain, a double center-deposited Ti chain, and a double edge-deposited Ti chain.

	Binding energy (eV/unit cell)	Magnetic moment (μ_B_)	
Ti1 (center)	−6.74	3.98	Half metal (majority spin)
Ti2 (center)	−15.93	4.01	Half metal (majority spin)
Ti2 (edge)	−17.40	4.00	Half metal (majority spin)

Adsorbed Ti atoms make the composite structure magnetic, gaining a total magnetic moment of 3.98 μ_B_ per unit cell. Distribution of spin density in Fig. 2(b) indicates that contributions to the magnetism derive from two sources: the adsorbed Ti atoms and the Mo atoms at the edges. Energy bands displayed in Fig. 2(c) clearly demonstrate that Ti 3*d* orbitals contribute almost exclusively to valence bands of both spins within 0.5 eV of the Fermi level. Above the Fermi level, Mo edge states appear in the conduction bands and mingle with the Ti impurity states in several places. Also shown in the second panel of Fig. 2(c) a single band carrying the majority spin crosses the Fermi level and is solely responsible for spin-polarized conduction of the structure. The band consists mostly of Ti and Mo 3*d* orbitals at both edges, with the Ti contribution represented by the size of red circles. Inset in the first panel concentrates on the evolution of the weight of the Ti orbitals in the band for the majority spin between −0.1 and 0.1 eV. It is evident that the contribution of Ti atoms drops considerably as the valence band moves close to the Fermi level, recovers substantially as the band shifts above the level, and finally shrinks again to almost zero as it approaches the Г point. Density of states (DOS) corresponding to the majority spin clearly illustrates this combination of Ti and edge Mo orbitals. DOS at the Fermi level, for example, comes from the Ti (red curve) and Mo at both edges (represented by green and blue curves).

The single band transport is therefore the result of the interaction between the deposited Ti chain and the edge Mo atoms. This interaction is also present in other conduction bands for both spins. Overall, an energy gap of 0.156 eV in bands of the minority spin straddling the Fermi level guarantees that the structure is a half metal.

**Figure 3:**
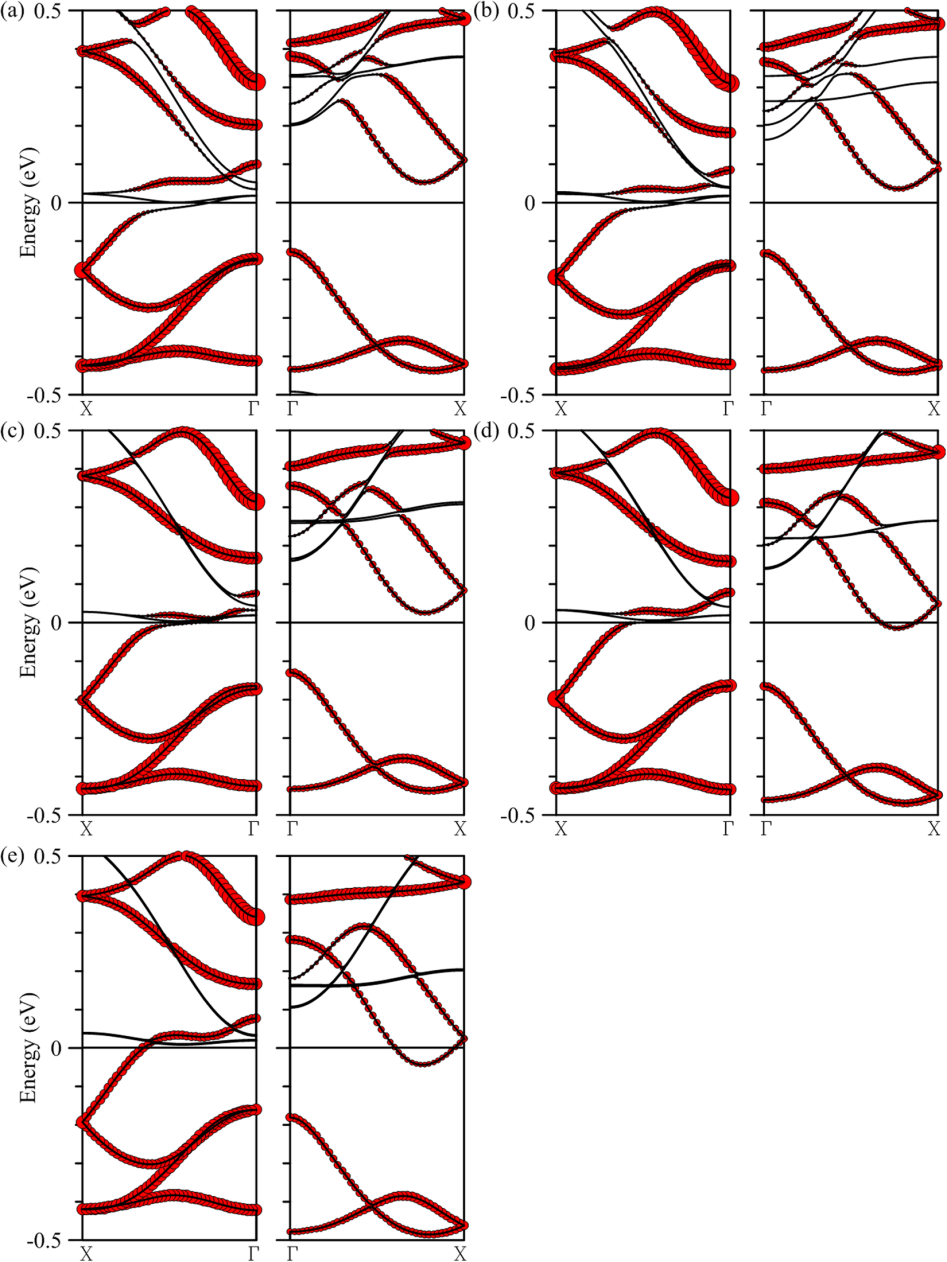
Energy bands of a single Ti chain deposited in the middle of a *N*a-MoS2 armchair nanoribbon, with *N*a equal to (**a**) 12, (**b**) 13, (**c**) 14, (**d**) 16, and (**e**) 20. The left (right) panel is for the majority (minority) spin. Contributions of Ti atoms are marked by the size of red circles.

Hybridization of Ti and edge Mo orbitals depends on the width of the MoS_2_ nanoribbon. For *N*a less than 15, interaction between Ti and edge Mo atoms stay strong and a single hybridized band continues to make the structure a 100% spin-polarized conductor. Shown in Fig. 3(a–c) are energy bands corresponding to *N*a = 12, 13, and 14 respectively. As the ribbon narrows the composition of the band crossing the Fermi level also changes, turning from chain-heavy to edge-heavy transport carrying only the majority spin. For *N*a larger than 15, as shown in Fig. 3(d,e) for *N*a = 16 and 20 respectively, a band for the minority spin shifts downward in energy through the Fermi level and terminates the half-metallic property. It is also apparent that as the ribbon becomes wider, hybridization of Ti and edge Mo orbitals diminishes as a result. Starting from *N*a = 20, energy bands are essentially the independent combination of the Ti and edge Mo bands. However, half-metallic property can be recovered if thicker Ti chains are used. In the case of 20-MoS_2_ armchair nanoribbon, for example, half metallicity is restored with a Ti double chain deposited in its middle, as is illustrated in Part II of Supplementary Information.

**Figure 4:**
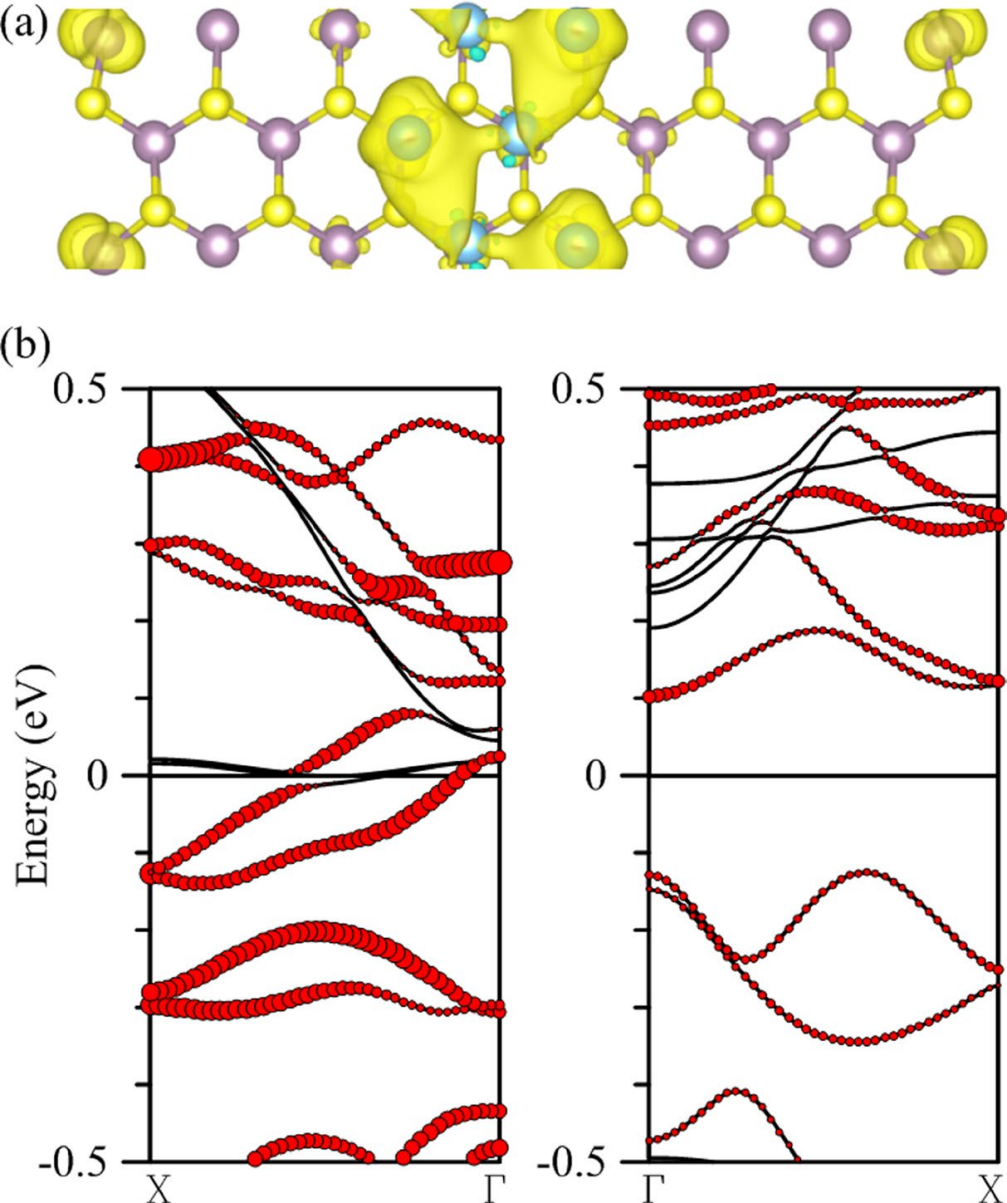
(**a**) Configuration and spin density of a double chain of Ti atoms deposited in the middle of the 15-MoS_2_ armchair nanoribbon. (**b**) Band structure of the composite, with the left (right) panel for the majority (minority) spin. Contributions of Ti atoms are marked by the size of red circles.

Half metallicity of 15-MoS_2_ can also be achieved by deposition of more Ti chains. In Fig. 4(a) a fully relaxed double chain having four Ti atoms per unit cell is deposited in the middle of the 15-MoS_2_ ribbon, with Ti atoms still settling down directly over Mo atoms. Increased adsorption of Ti atoms actually strengthens their binding from −3.27 eV (or −6.74 eV per unit cell) to −3.98 eV (or −15.93 eV per unit cell) for each Ti atom. Spin density concentrates on Ti and edge Mo atoms as before, but total magnetic moment changes little. There are now two bands crossing the Fermi level for the majority spin (left panel of Fig. 4(b)), which again comes from the contributions of Ti (red circles) and Mo atoms at both edges. A wider band gap about 0.227 eV is also present for bands in the minority spin.

**Figure 5:**
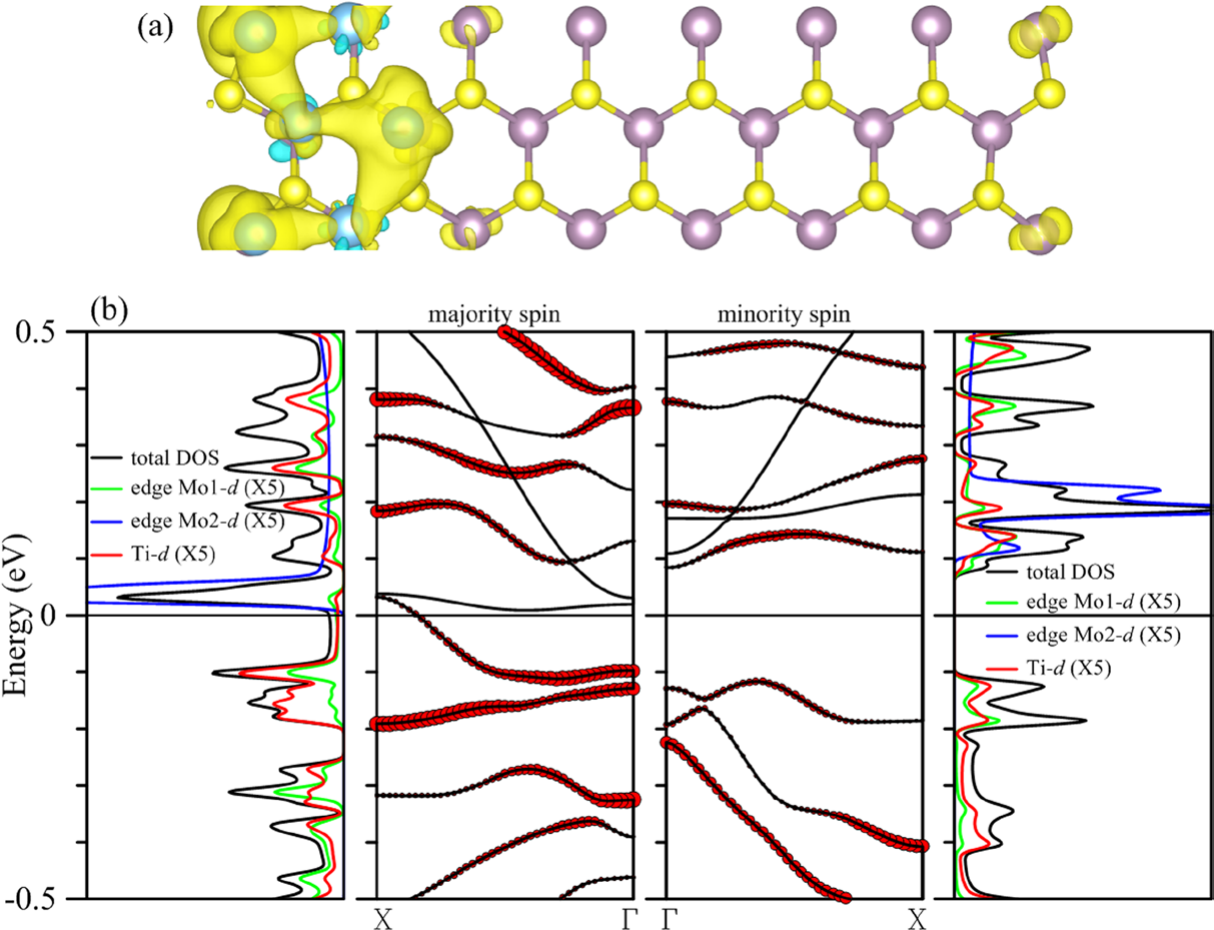
(**a**) Configuration and spin density of a double chain of Ti atoms deposited at the left edge of the 15-MoS_2_ armchair nanoribbon. (**b**) The left (right) two panels are DOS and energy bands corresponding to the majority (minority) spin. Local DOS of individual Ti and Mo atoms at both edges are magnified five times for clarity, with the green curve representing the Mo atoms at the edge where Ti atoms are adsorbed. Contributions of Ti atoms are marked by the size of red circles in the energy bands.

In another possible scenario in which the double chain of Ti atoms is deposited at one end of the ribbon as shown in Fig. 5(a), calculation indicates that the binding is even stronger, reaching −4.35 eV per Ti atom, and the magnetic moment is 4.00 μ_B_ per unit cell. Band structure in Fig. 5(b) again reveal a gap about 0.200 eV separating the valence bands from conductions bands for the minority spin, and a single band having the majority spin crossing the Fermi level. The band is made of Ti and Mo *d* orbitals at the very edge where deposition occurs, which also means that Mo atoms at the other end of the ribbon do not participate in the spin-polarized conduction. Due to much longer separation between the Ti chain at one end and the Mo atoms at the other end, their electron wavefunctions overlap negligibly and therefore do not hybridize to become part of the crucial band. The second panel of Fig. 5(b) also shows that the contribution from Ti orbitals to the band crossing the Fermi level decreases monotonically all the way from the Г point to the Brillouin zone boundary. DOS assists the identification of major contributors. The blue curve of the first panel illustrates the contribution from the Mo *d* orbitals at the edge without Ti deposition. It has a spike right above the Fermi level, deriving from the relatively flat band and another band having more dispersion in the second panel, with both bands free of Ti atom contribution. DOS for the same Mo atoms at the far end is also plotted by blue curve in the fourth panel, which is easily connected to two bands for the minority spin above the Fermi level.

**Figure 6:**
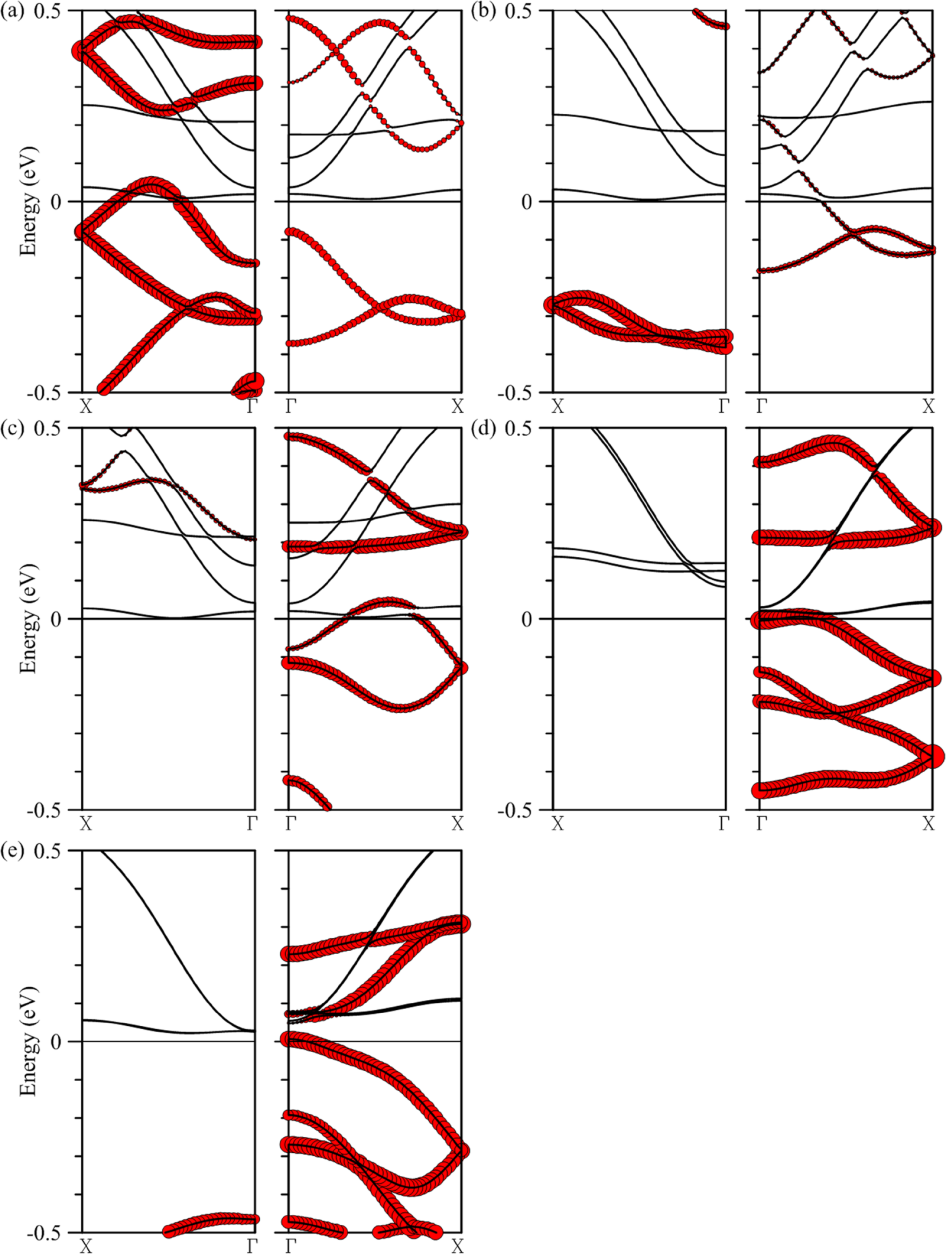
Energy bands of a single (**a**) V, (**b**) Cr, (**c**) Mn, (**d**) Fe, and (**e**) Co chain deposited in the middle of a 15-MoS2 armchair nanoribbon. Left (right) panels are for the majority (minority) spin. Contributions of Ti atoms are marked by the size of red circles.

It is also possible to transform the armchair MoS_2_ nanoribbon into a half metal by adsorption of other transition metals. Listed in Table 2 are calculation results of single atomic chains made of V, Cr, Mn, Fe, and Co deposited in the middle of the 15-MoS_2_ ribbon individually. Binding energy turns weaker from V to Cr first, bouncing back, however, from Mn to Co. But even for the least attached Cr chain the binding is still close to −3 eV per unit cell. Large magnetic moment is also produced in each composite structure, with the largest being associated with the Cr chain. Shown in Fig. 6 are band structures for the five composites, with the red circles representing contributions from adsorbed atomic chains. It is also noticeable that, except for the case of V chain adsorption, half metallicity occurs in bands associated with the minority spin. Bands crossing the Fermi level can be variably modulated by the Mo atoms at the ribbon edges as in the cases of V, Cr, and Mn, or are dominated more uniformly by the impurity atoms in the atomic chain as in Fe and Co and are thus quite independent of the edge states.

**Table 2:** Binding energy and magnetic moment per unit cell for a 15-MoS_2_ armchair nanoribbon deposited with a single transition-metal chain or Si chain.

	Binding energy (eV/unit cell)	Magnetic moment (μ_B_)	
V	−5.59	5.62	Half metal (majority spin)
Cr	−2.99	8.42	Half metal (minority spin)
Mn	−3.16	6.48	Half metal (minority spin)
Fe	−4.93	4.00	Half metal (minority spin)
Co	−6.38	2.21	Half metal (minority spin)
Si	−4.67	0.00	Half metal (minority spin)

**Figure 7:**
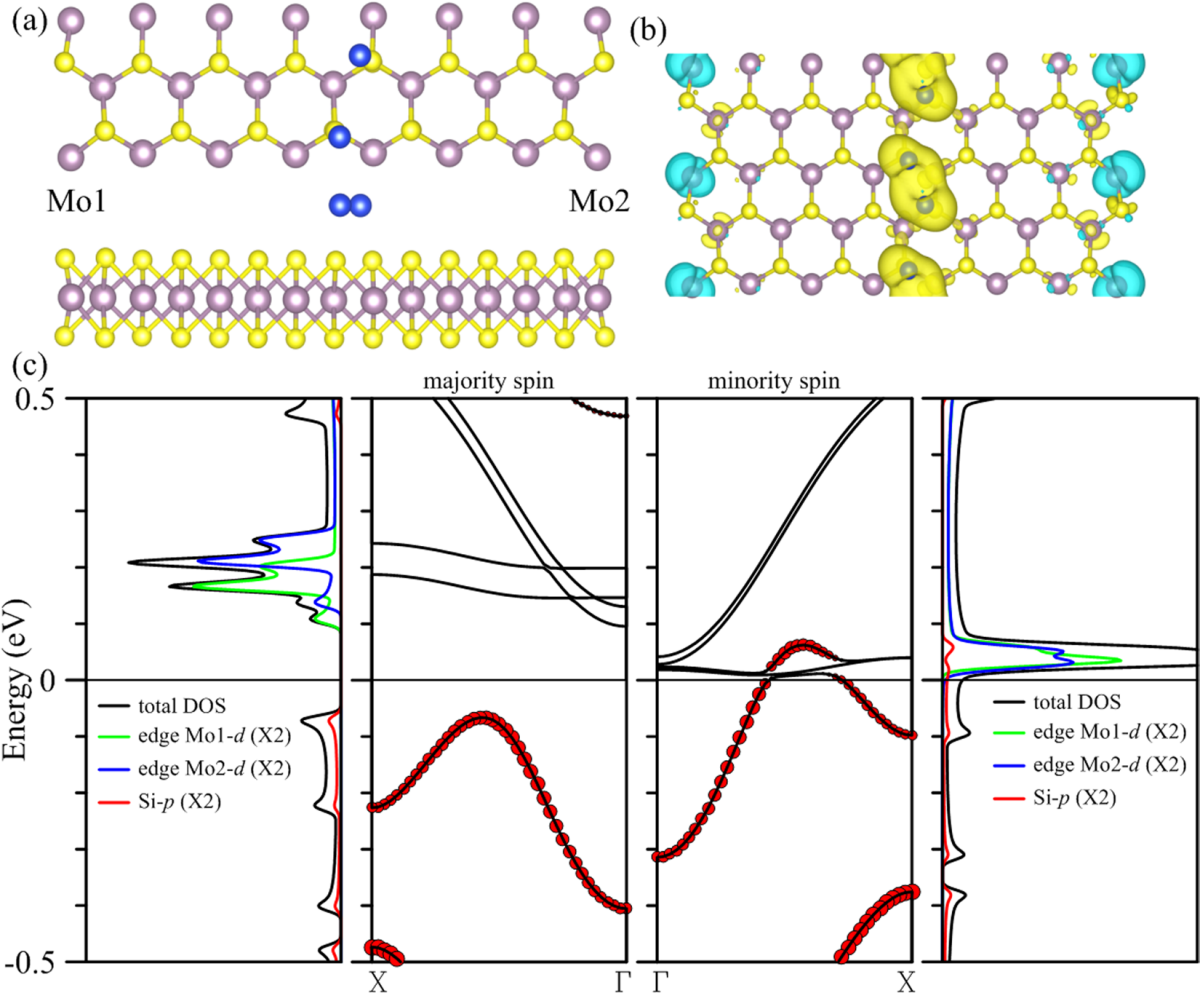
(**a**) Configuration of a single chain of Si atoms (blue spheres) deposited in the middle of the 15-MoS_2_ armchair nanoribbon, and (**b**) spin density of the composite. (**c**) The left (right) two panels are DOS and energy bands corresponding to the majority (minority) spin. Local DOS of single Si and Mo atoms at both edges are magnified twice as large for clarity. Contributions of Si atoms are marked by the size of red circles in the energy bands.

Lastly we report the deposition of a single Si chain on 15-MoS_2_. Figure 7(a) depicts a configuration quite unlike those with transition-metal deposition. A fully relaxed structure now has the positions of Si atoms higher in the z direction than the S atoms but has stronger binding than Cr and Mn. The S-Si distance ranges between 2.26 and 2.29 Å. The structure has a zero total moment but antiferromagnetic distribution of spin density as shown in Fig. 7(b), with the Si chain carrying the majority spin and the edge Mo atoms the minority spin. One important property in common with previous cases is that the interaction between the adsorbed impurity chain and edge Mo atoms remains the decisive factor for the conduction. In Fig. 7(c) a band of the minority spin crosses the Fermi level as the contribution of *p* orbitals from the Si atoms diminishes and that of *d* orbitals from edge Mo atoms begins to dominate. A gap, however, is opened between the valence and conduction bands for the majority spin. DOS in the fourth panel clearly indicates that Si *p* orbitals and Mo *d* orbitals at both ends of the ribbon hybridize to make the composite a half metal.

In summary, we have used first-principles calculations to demonstrate that armchair MoS_2_ nanoribbons can be transformed into half metals by adsorbing a variety of transition-metal chains. The interaction of deposited transition-metal chain with the Mo atoms at the edges of the ribbon is the major factor in creating completely spin-polarized conduction in some of the composites, and in others the transition metals alone are enough to achieve half metallicity. We also show that a Si atomic chain with its *p*-*d* hybridization with the edge Mo atoms is equally capable of providing a perfect spin-polarized channel. These results should be useful for designing and creating nanoscale conduits in spintronic applications.

## Methods

In the derivation of the optimal structures and energy bands, we performed spin-polarized density functional calculation using the VASP code^21,22^. Projector augmented-wave (PAW) pseudopotentials and the exchange-correlation functionals of Perdew, Burke and Ernzerhof (PBE)^23^ were chosen to execute the calculation. Multiple k-point sampling in the first Brillouin zone and a height no less than 15 Å perpendicular to the ribbon in the supercell is allocated for vacuum space to eliminate artificial interaction between the cells. Cut-off energies for the expansion of wave functions and potentials in the plane-wave basis were no less than 350 eV.

## Electronic supplementary material

Supplementary information
