# Supplementary material for: Armchair MoS2 nanoribbons turned into half metals through deposition of transition-metal and Si atomic chains
Source: Sci Rep. 2018 Sep 6;8:13307. doi: 10.1038/s41598-018-31684-z (PMC6127190; doi:10.1038/s41598-018-31684-z)
Supplement: Supplementary file 1 — Supplementary information [file 41598_2018_31684_MOESM1_ESM.pdf]

# Supplementary information for the article: Armchair $MoS_2$ nanoribbons turned into half metals through deposition of transition-metal and Si atomic chains

Chi-Hsuan Lee, Joy Lin, and Chih-Kai Yang*

Graduate Institute of Applied Physics, National Chengchi University, Taipei 11605, Taiwan, ROC.

## I. A zigzag 8-$MoS_2$ nanoribbon deposited with a single Ti chain

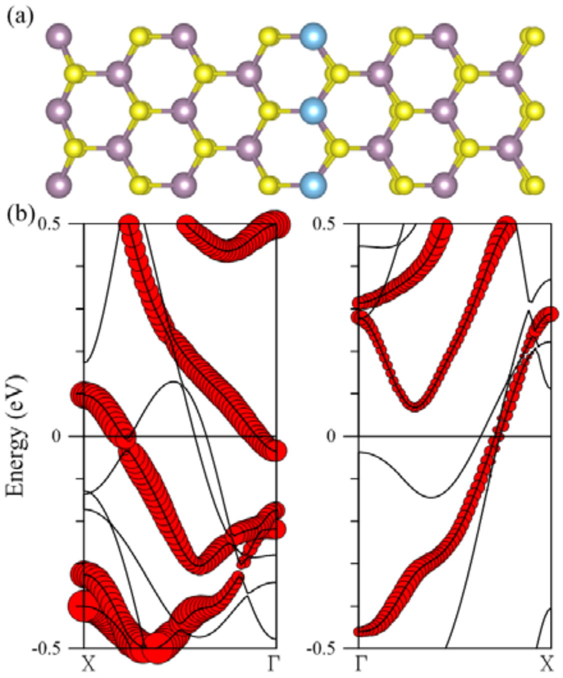


Relaxed configuration of an 8-$MoS_2$ zigzag nanoribbon deposited with a single Ti chain (azure spheres) is represented in the figure. Band structure indicates that conduction is possible for electrons with either spin. Contributions of Ti atoms are marked by the size of red circles

## II. A 20-$MoS_2$ armchair nanoribbon turned into half metal

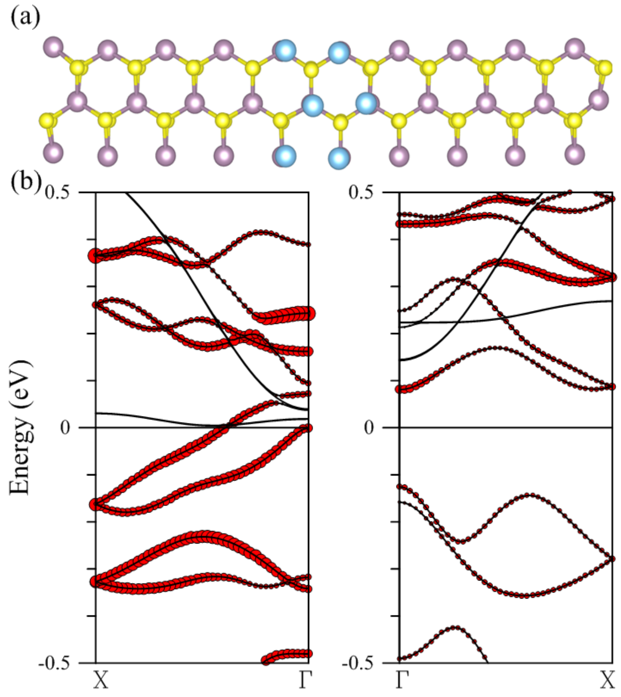


Although the 20-$MoS_2$ nanoribbon with an adsorbed single Ti-chain is not half metallic, a double Ti-chain adsorption can increase the interaction between Ti and edge Mo atoms and transform the composite into a half metal.

## III. Another configuration for an armchair 15-$MoS_2$ nanoribbon with an adsorbed Ti chain

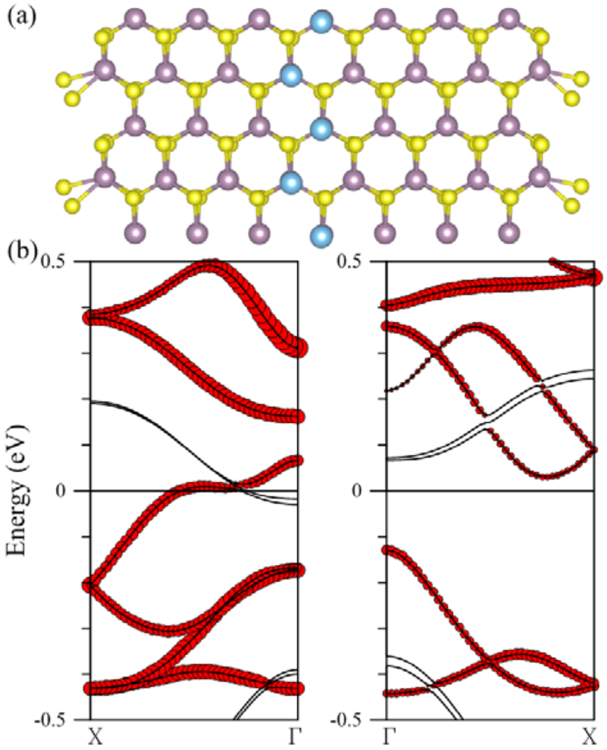


With the edge Mo atoms passivated by S, the configuration is one possible configuration for a 15-$MoS_2$ armchair nanoribbon deposited with a single chain of Ti atoms. Band structure shows that this composite is also half metallic.
